# Molecularly Imprinted Polymers (MIPs) in Sensors for Environmental and Biomedical Applications: A Review

**DOI:** 10.3390/molecules26206233

**Published:** 2021-10-15

**Authors:** Abbas J. Kadhem, Guillermina J. Gentile, Maria M. Fidalgo de Cortalezzi

**Affiliations:** 1 Department of Civil and Environmental Engineering, University of Missouri, E2509 Lafferre Hall, Columbia, MO 65211, USA; ajkqmb@mail.missouri.edu; 2 Department of Chemical Engineering, Instituto Tecnológico de Buenos Aires, Lavardén 315, Buenos Aires C1437FBG, Argentina; ggentile@itba.edu.ar

**Keywords:** molecular imprinted polymers, environmental sensing, biomedical devices

## Abstract

Molecular imprinted polymers are custom made materials with specific recognition sites for a target molecule. Their specificity and the variety of materials and physical shapes in which they can be fabricated make them ideal components for sensing platforms. Despite their excellent properties, MIP-based sensors have rarely left the academic laboratory environment. This work presents a comprehensive review of recent reports in the environmental and biomedical fields, with a focus on electrochemical and optical signaling mechanisms. The discussion aims to identify knowledge gaps that hinder the translation of MIP-based technology from research laboratories to commercialization.

## 1. Introduction

The mechanism for the specific recognition of antibodies and antigens, enzymes and substrates, hormones and receptors inspired the development of synthetic materials that mimic nature’s ability to selectively capture chemical species from complex mixtures [1]. Molecularly imprinted materials are tailor-made polymers that present molecular recognition sites for a specific or closely-related target molecule [2]. Prior to polymerization, the target analyte, or template, is combined with a functional monomer to form a precursor structure by covalent [3], semi-covalent [4], or non-covalent [5,6] bonding. Then, they are polymerized in the presence of a crosslinker, along with an initiator in a porogenic solvent. Afterwards, the template is eluted, by extraction with a proper solvent or by chemical cleavage, to create empty recognition cavities in the polymer matrix, whose morphology and functionality are complementary to those of the template molecule [7,8].

The concept of molecular imprinting dates from 1930, but it was not until the description made by Wulff and Sarhan in 1972 [9] that research on molecularly imprinted polymers (MIPs) attracted scientific interest, driven by their promising characteristics: simplicity, robustness, stability, ease of preparation, and high affinity and selectivity towards the target molecule [10,11,12,13].

MIPs have been fabricated for solid phase extraction [14,15,16,17,18], chromatographic separation [19,20,21,22,23], catalysis [24,25,26,27,28], drug delivery [29,30,31,32,33], study of the structure and function of proteins [34,35,36,37,38], environmental and biomedical sensing [39,40,41,42,43], water and wastewater treatment [44,45,46,47,48], and membrane-based separations [49,50,51,52,53]. MIP use for purification purposes is the most commercially available application, particularly in analytical chemistry; other uses are still in need of further development [54].

The extensive literature on MIPs for sensing applications comprises a wide variety of fields. The transformative impact of MIP-based sensing for environmental and biomedical application is associated with their potential capacity to detect compounds at trace levels in complex matrices without pretreatment, which would open possibilities for contaminant monitoring in situ, as well as fast clinical analysis at the point of care for improved diagnosis and treatment. However, and although there is a genuine market need for such devices, MIP-based technology has remained mostly in the academic field.

This article aims to review advances in imprinted molecular technologies, particularly those applied to sensors in the environmental and biomedical fields. First, the most commonly used polymerization methods, physical forms, and materials are briefly described, followed by a comprehensive review of sensor fabrication reports of electrochemical and optical sensors. Given the simplicity and widespread availability of instruments for the detection of electrical and optical signals, these two mechanisms are the most promising for in situ testing and point of care diagnosis. Selected research is described in more detail for each mode of operation and application, to identify knowledge gaps and hurdles in the transition of the technology from laboratory development to commercial products.

## 2. Synthesis

**Figure 1:**
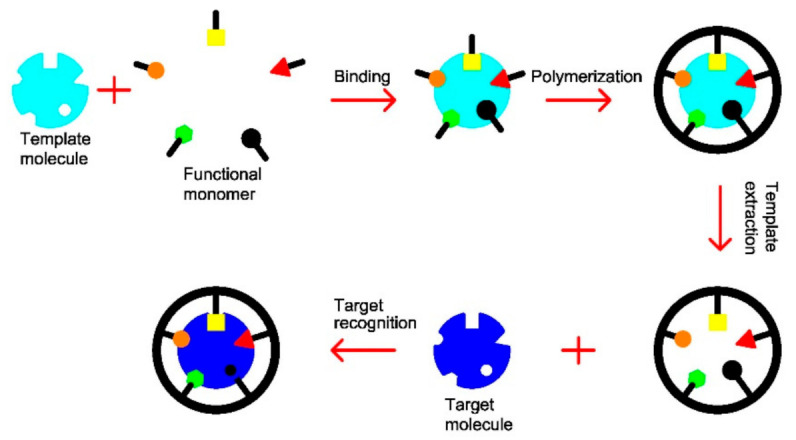
Schematic diagram of the molecularly imprinting process.

In the synthesis process, the template molecule is covalently or non-covalently reversibly bonded to the functional monomer, with appropriate binding groups, and then polymerized with an excess of crosslinker [55]. The subsequent removal of the template originates microcavities, which are complementary to the shape, size, and spatially orientated functional groups of the template molecule [1,10]. Figure 1 presents a scheme of the imprinting process.

The functional monomer confers chemical stability before and during the polymerization and provides the MIP with the ability to interact with the target molecule, while the crosslinker offers mechanical stability and adequate porosity to the polymer, stabilizes the recognition sites, and determines the hydrophobicity [2]. A porogenic solvent brings all components into a homogenous system during the polymerization and creates the pores in the structure [56].

The choice of the functional monomer depends on the chemical structure of the template molecule and on the medium for which the MIP is designed (aqueous or organic). In environmental and biomedical applications, affinity with aqueous solutions is required, limiting the selection to hydrophilic materials. Strong template-monomer interactions enhance the ulterior affinity between the target analyte and the recognition sites. The molecule of the monomer has two units, one for recognition and the other for the polymerization. MIPs constituted by only one monomer have no more than two different kinds of binding interactions, which is sufficient for small molecules [57]. Instead, larger molecules with many functional groups require different specific bonds to achieve a desirable affinity and to prevent non-specific binding; thus, a combination of monomers may be selected: a neutral one as the backbone, along with another that is charged, hydrophobic, or capable of developing hydrogen bonds for constructing the imprinted cavities [58]. Biologically functional molecules that can specifically bind to the template molecule may also be combined with the monomer for enhancing selectivity and affinity; such is the case of aptamers, single-stranded oligonucleotides, or peptides with the ability to bind to proteins and nucleic acids [57].

The covalent imprinting route requires the formation of covalent bonds between the functional monomer and the template before polymerization, as well as between the MIP and the target molecule [59]. These bonds should be stable during the polymerization process and be cleaved without harming the MIP. The non-covalent approach, on the other hand, relies principally on hydrogen bonds, but also on hydrophobic, electrostatic, dipole-dipole, and ionic interactions between the functional monomer and the template and between the MIP and the target [60]. The non-covalent method is the most frequently used, due to ease of both preparation and template removal (by a simple wash in acidic or basic aqueous solution or with alcohol), as well as fast rebinding (with the target molecule) kinetics [61]. However, two limitations may arise: first, if the MIP is placed in a polar solvent, the interactions between the template and the functional monomer can be easily disrupted; and second, if the target molecule has only one point of interaction, the recognition properties are limited [59]. These limitations can be partially overcome in the semi-covalent imprinting, in which covalent bonds are formed between the monomer and the template and non-covalent interactions arise between the MIP and the analyte [4,62].

Covalent bonds lead to a sole organization of the functional groups in the cavities, in terms of number and orientation, whereas weaker non-covalent interactions result in less selectivity, since the target may enter the cavity in incorrect orientations, making it necessary to create an excess of binding sites to achieve the required orientation [55]. The template stoichiometrically attaches to the functional monomer in the covalent approach. Instead, the ratio of template to functional monomer usually used is 1:1 to 1:2 for the semi-covalent technique and 1:4 to 1:8 for the non-covalent, according to the affinity between them and the complexity of the template molecule [2].

Conductive polymers can be fabricated by chemical or electrochemical routes from aqueous solutions of their monomers, including enzymatic routes [63]. The imprinted sites are created based on the irreversible overoxidation that the polymers undergo during and after polymerization; the target is expelled from the polymer due to the overoxidation and, therefore, the template extraction procedure and its related complications are avoided [64]. Oxidative-chemical polymerization has been widely used due to its simplicity [63]. It is initiated by an oxidizing compound, such as FeCl_3_ or H_2_O_2_ [63], and is applied to the synthesis of polypyrrole, polyaniline, polythiophene, poly(1,10-phenanthroline-5,6-dione), poly(pyrrole-2-carboxylic acid), poly-9,10-phenanthrenequinone, polyphenanthroline, and some other conducting polymers. The most important electrochemical method of preparing conducting polymers is the anodic oxidation of suitable monomer species when the polymer formation and oxidation processes occur simultaneously [65]. Electrochemical polymerization has advantages over the chemical methods, as the overoxidation of the polymer creates oxygen containing groups that are useful to promote the recognition/attachment of the MIP target compounds [63]. On the other hand, cathodic electropolymerization has rarely been applied to the synthesis of conducting polymers [65]. Some redox enzymes (oxidases, such as glucose oxidase) and their substrates were used in a process similar to the chemical polymerization, due to their catalytic action that forms hydrogen peroxide. This process is conducted in an aqueous environment at neutral pH and room temperature, for maximal enzymatic activity, which, in turn, results in high biocompatibility of the polymers as desirable for biosensing applications [63]. Polymer deposition can be achieved by nucleation, growth, and other chemical steps in solid state conditions applying potentiostatic, potentiodynamic, or galvanostatic techniques to start and control these processes [65]. The selection of the deposition technique and the adjustment of the process parameters enable the formation of sensors with different characteristics. The process parameters most commonly adjusted are the applied voltage, potential pulse duration or potential sweep rate (cycling), and the electrical current [63].

### 2.1. Imprinting Techniques for Sensors

Ertürk and Mattiasson [66] describe bulk, epitope, and surface imprinting techniques that are especially used in the field of sensors.

Bulk imprinting requires the whole template molecule to be fully imprinted in the polymeric matrix, followed by polymerization and, finally, template removal. Then, the bulk polymer is crushed to obtain smaller particles. This is the preferred option in the case of small templates, since adsorption and release of the molecule are faster and reversible, with the consequent option of support reuse.

Epitope imprinting, on the other hand, relies on a small part of the template molecule being imprinted, making it useful for macromolecules, where only the imprinted fraction is able to represent the whole molecule, and reducing non-specific binding. Rachkov and Minoura [67] employed this concept to covalently bond only a peptide epitope, instead of the whole protein, to a surface, prior to polymerization. Removal of the support surface with the template allowed a thin film to be obtained, which proved effective in capturing the target protein.

Surface imprinting produces recognition sites only on the surface of the substrate by different routes, i.e., soft lithography, template immobilization, grafting, and emulsion polymerization. As the imprinting only takes place on the surface, a lower amount of template molecules is required, but sensitivity decreases because of the reduction in imprinting sites.

Other techniques have been applied less frequently to MIP fabrication, including soft lithography, template immobilization, and grafting.

Soft lithography (stamping) includes a step in which a pre-polymerization layer is coated on a transducer surface and where the template stamp is pressed. The obtained films are complementary to the template in structure, geometry, and chemistry.

Template immobilization is based on a molecule that is fixed onto a solid support by chemical bondings. For example, proteins are adsorbed on a support and are surrounded by the formation of a layer of disaccharides, followed by the formation of a thin plasma film. Dissolution and extraction of the template protein produce cavities on the surface that are complementary to the protein. Grafting first adsorbs the template to a support already grafted with the polymeric functional groups, obtaining affinity for the target.

### 2.2. Materials for MIP Fabrication

MIPs have been reported in a wide variety of functional monomers and crosslinkers, mainly determined by the type of application and target intended, and the initiators given by the polymerization reaction route.

Among the functional monomers, those containing carboxyl groups are preferred for the non-covalent technique, since they can be hydrogen donors and acceptors at the same time and are useful for the formation of hydrogen bonds. For instance, methacrylic acid (MAA) participates in non-covalent imprinting [54,57,58,60,68,69,70], forming ionic unions with amines and hydrogen bonds with amides, carbamates, and carboxylic groups [69]. Other functional monomers often used in the non-covalent technique include acrylamide (AAM) [54,58,60,70], acrylic acid (AA) [58,60,69], methyl methacrylate [54,69], 4-vinylpyridine [69,70], 2-vinylpyridine [69], and 1-vinylimidazole [69]. Pyrrole [54,68,71] offers good electrical conductivity, biocompatibility, and redox activity [72]. Aniline [54] forms polyaniline, a conducting polymer with a reversible redox system, environmental stability, and easy chemical or electrochemical polymerization [73]. In an extensive work, Chen et al. [61] mentioned the less often employed 2-acrylamido-2-methyl-1-propane sulfonic acid, 3-aminopropyltriethoxysilane (APTES), 4-ethylstyrene, glycidoxypropyltrimethoxysilane, 2-hydroxyethyl methacrylate (HEMA), itaconic acid (IA), methacrylamide (MAAM), 3-methylacryloxyprolyl trimethoxysilane, methylvinyldiethoxysilane, styrene, p-Vinylbenzoic acid, trans-3-(3-pyridyl)-acrylic acid, and trifluoromethyl acrylic acid. Otherwise, *N*-isopropylacrylamide (NIPAAm) [58,60,74], and o-phenylenediamine (o-PD) [75,76] may also be cited for the non-covalent approach. Vinylferrocene and ferrocenylmethyl methacrylate have been used as electroactive monomers, which allowed for the electrochemical detection of non-electroactive targets [77,78,79,80]. Fewer monomers can be selected for the semi-covalent or covalent approach, since specific bonds must develop; examples of these functional monomers are tert-butyl p-vinylphenol carbonate, 4-vinyl aniline, 4-vinyl benzaldehyde, and 4-vinyl benzene boric acid for covalent [61], whereas 3-isocyanatopropyltriethoxysilane [61] and m-aminophenylboronic acid [60,81] have been used for semi-covalent imprinting, although it is also highly dependent on the target molecule’s chemistry and interactions.

The two most often used crosslinkers are ethylene glycol dimethacrylate (EGDMA) [8,54,57,60,61,69] and divinylbenzene [8,60,61,69]. Both chemicals were applied in the non-covalent approach by means of free radical polymerization. Other compounds commonly reviewed for the same purpose are 3,5-bis(acryloylamido)benzoic acid, *N,O*-bisacryloyl-*L*-phenylalaninol, *N,O*-bis methacryloyl ethanolamine, pentaerythritol triacrylate, trimethylolpropane trimethacrylate [61,69], 2,6-bisacryloylamidopyridine, 1,4-diacryloyl piperazine, glycidil methacrylate, 1,3-diisopropenyl benzene, *N,N’*-methylenediacrylamide, *N,N’*-1,4-phenylenediacrylamine, and tetramethylene dimethacrylate [61]. For the free-radical polymerization of covalent complexes, bis-(1-(tert-butylperoxy)-1-methylethyl)-benzene, dicumyl peroxide, and triallyl isocyanurate have been mentioned [61]. Also, *N,N’*-methylenebisacrylamide (MBA) [8,60,69], *N,N*-1,4-phenylenediacrylamide, and pentaerythritol tetraacrylate [69] were found as common examples of crosslinkers.

In free radical polymerization, the pre-polymerization monomer-target complex is subjected to heat or ultraviolet (UV) radiation in the presence of an initiator, such as 2,2′-azobis(isobutyronitrile) (AIBN) [54,61], 4,4′-azo(4-cyanovaleric acid), or azobis dimethylvaleronitrile [61]. UV light at the maximum absorption wavelength of the compound or high temperature enables the decomposition of the azo compounds and generates free radicals that start the chain reactions. Organic peroxides, e.g., benzoyl peroxide or benzyl dimethyl acetal, initiate the process by redox reactions, heat, or photochemically, and are especially suitable for aqueous matrices, since they are soluble in water as well as in organic solvents. Inorganic oxidants, such as potassium persulfate, have also been reported.

For the detection of organic, relatively hydrophobic pollutants in environmental applications, the porogenic solvent tends to be aprotic and non-polar, e.g., chloroform, toluene, acetonitrile, and tetrahydrofuran [59]. However, biomolecules require other kinds of solvents, since they are usually insoluble or unstable in organics. The polarity of the porogen is a key characteristic for its selection, particularly in non-covalent MIPs, as it affects the interactions between the template and the functional monomer, which, in turn, define the adsorption properties.

### 2.3. Physical Form

**Figure 2:**
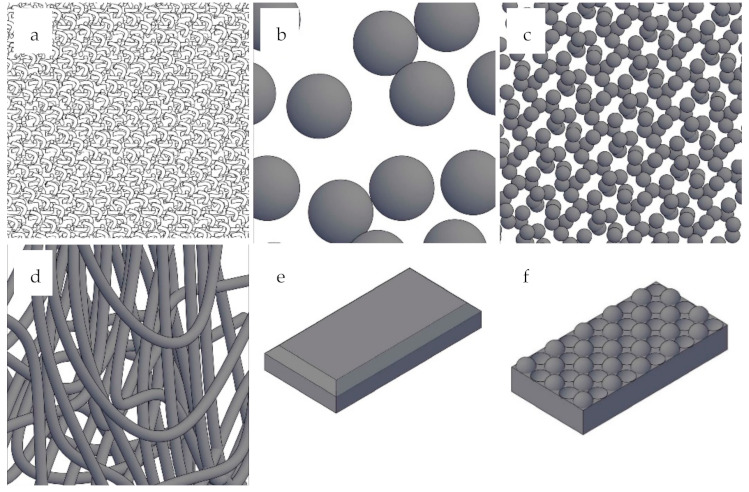
MIP physical forms: (**a**) blocks or monoliths, (**b**) microspheres, (**c**) nanospheres, (**d**) thin films, (**e**) nanocomposite membranes, and (**f**) nanowires.

MIPs have been reported in different physical forms, such as blocks or monoliths, microspheres, nanospheres, thin films, nanocomposite membranes, and nanowires. Figure 2 shows a schematic illustration of these physical forms.

The choice of the physical form is often dictated by the application, and it defines the polymerization technique to be used [82].

Nano and microspheres have both surface and internal porosity, which results in desirable properties such as high surface area, low density, and a high loading capacity, and may find applications in analytical chemistry and biomedical applications: drug delivery, absorption and desorption, high speed chromatography, and tissue regeneration [83,84].

Different polymerization techniques may produce porous microspheres, such as suspension [85,86,87,88], precipitation [89,90,91,92,93], emulsion [94,95,96,97], grafting [98,99], and swelling [100,101,102], as well as a combination of two of these methods [103,104]. For example, in swelling polymerization, previously formed beaded seeds made of silica or polymers are used as scaffolds for the polymerization.

The suspension polymerization process is useful when both the monomer and the crosslinker are insoluble in the porogen, thus forming two phases, i.e., a liquid matrix that contains droplets of the monomer, inside which polymerization occurs [83]. The obtained spheres usually have a diameter between 10 and 100 μm [105]. It is often necessary to include an adequate stabilizer that covers the droplets in the form of a thin film and prevents coalescence, controling the size of the particles [106]. Common stabilizers include surfactants [107] and ionic liquids [108]. Solvents compatible with the stabilizers may be water [109], silicone oil [110], and polyvinyl alcohol [111]. As stated above, water may interfere with the non-covalent bonding between the functional monomer and the template; therefore, its use is limited to those cases where the stability is not affected, while the other solvents mentioned were able to overcome this drawback, but the monodispersivity was negatively affected [112].

In the precipitation or dispersion polymerization process, the components of the MIP are mixed with a much higher amount of solvent than required in the traditional polymerization. The polymer grows at a larger extent and precipitates when the chain is long enough to be insoluble [113]. The obtained particles are smaller, usually between 0.3 and 10 μm [114], with more uniform size, and are recovered by simple washing and centrifugation. It is possible to control the size and shape of the particles [115] without the aid of surfactants or stabilizers, which avoids potential contamination of the MIPs. This technique was first developed by Mosbach and collaborators [116] for the synthesis of MIP microspheres [117], nanospheres [118], core-shell particles [119], thin films [120], and nanocomposites [121].

Core-shell MIP particles are obtained by grafting or surface polymerization. All the MIP components are adsorbed on the surface of preformed beads, such as porous silica or spherical polymers, before the polymerization starts. Once the free-radical polymerization is over, the bead is removed, thus obtaining a spherical particle coated by a thin layer of MIP. It is important to limit the free-radical polymerization to the bead surface, for example, with the inclusion of an immobilized chemical compound whose functions are to initiate, transfer, and terminate the polymerization (iniferters) [122,123]. Advantages of the technique are a high surface density of polymer chains, high stability of the coated layer, and the ability to graft different polymers to the same substrate [124,125].

Emulsion polymerization can lead to both spherical and core-shell particles. The monomers are immersed in a solvent, usually water, in which they do not dissolve, and emulsification takes place. Then, the polymerization is started by an initiator soluble in one phase. When the process is finished, a fluid of milky consistency is obtained, which is usually referred as latex, synthetic latex, or polymer dispersion. In contrast with suspension polymerization, the droplets are inside micelles stabilized with the aid of a surfactant. To prepare core-shell particles, the core particles are produced and afterwards the shell layer is generated by emulsion polymerization. The type and amount of surfactant controls the particle size, usually in the range of 10–1000 nm [126,127]. The main disadvantage of this method is the use of water and surfactants that may precipitate interferences during polymerization between the template and the functional monomer, particularly in the non-covalent approach.

Block or monolith MIPs are obtained by bulk polymerization, also known as the traditional method. Bulk polymerization is the simplest and most widely used method, since the equipment is not sophisticated and no particular knowledge or mastery of organic chemistry is needed. The main components (template, functional, and cross-linking monomers) are mixed along with the initiator in a low volume of porogenic solvent, and the polymerization starts by heat or UV radiation. When the process is concluded, the monoliths are crushed, ground, and sieved to the desired particle size, reaching the micrometer range. Evident disadvantages of the grinding are that the obtained material is irregular in shape and size, nanosized particles cannot be produced, and many recognition sites are broken or inaccessible due to the lack of internal porosity.

In the field of analytical chemistry, Matsui et al. [128,129] introduced the monolithic imprinted polymerization technique that enables monolithic imprinted columns to be obtained for HPLC by a simple, one-step, free radical polymerization process that takes place within a chromatographic column. The template, functional monomer, cross-linking monomer, and initiator are first dissolved in the porogen and then the mixture is poured into the column. After the polymerization takes place, the template and the solvent are washed out of the column. An advantage of this route compared to traditional polymerization is that it is not necessary to crush, grind, sift, and pack the final material. Other advantages reported by the author are ease of preparation, good reproducibility, selectivity, sensitivity, high porosity, permeability, surface area, and fast mass transport.

## 3. Environmental and Biomedical Applications of MIPs

The specific binding properties of MIPs make them ideal materials for sensor fabrication. Although the literature on polymer formulation and synthesis methods is extensive, reports of effective use of MIPs on specific applications are scarce. Moreover, in order to use a MIP as a sensor, it needs to be coupled with a transducer or reading mechanism capable of determining the amount of target rebinding to the MIP after exposure to the test sample. The transducer technique should be simple, reliable, and not require external instruments or supplies. Based on these characteristics and their user-friendliness, this review focuses on electrochemical and optical sensors.

A shared objective of MIP-based sensors is to provide the market with simple, fast, and inexpensive methodologies for the detection and quantification of a chemical compound. Besides, researchers have focused their efforts on avoiding sample pretreatments, which are often time-consuming, tedious, or expensive, and in many cases involve toxic or hazardous solvents. Current protocols typically include a first pretreatment step, where the target of interest is removed from a complex matrix (e.g., by liquid extraction), followed by separation (e.g., by gas or liquid chromatography), enrichment, and detection.

A comprehensive review of environmental and biomedical sensors is presented below, organized by the sensing mechanism: electrochemical or optical. The limit of detection (LOD) and the limit of quantification (LOQ) are presented for each work, when reported by the authors and calculated as three and ten times the standard deviation, respectively, of the blank measured with the standards divided by the slope of calibration curve, as suggested by MacDougall et al. [130]. The units given by the authors were reproduced in this work to provide understandable and useful information, according to the specific target. Target molecules and the matrix in which they were tested are also listed for each work. Although all the cited reports focus on environmental or biomedical applications, not all of them employ a testing matrix that mimics real natural environments or the actual subjects of study (humans or animals). The advances and discoveries in the field are impressive and promising for the technology to transfer outside of research laboratories to commercial products. Yet, some shortcomings that need to be addressed are: limited detection, slow response, high LODs and LOQs, interferences, impaired performance in real complex mixtures, and reusability of the adsorbent to reduce waste, as well as devising, conceiving, and putting to work platforms to manufacture ready-to-use devices.

### 3.1. Electrochemical Sensors in Environmental and Biomedical Applications

Electrochemical sensors include a cell with a working electrode of particular interest, accompanied by a reference and an auxiliary electrode. Depending on the measured electrical parameter, they can be classified into three categories: conductivity or capacitance sensors, potentiometric sensors, and voltammetric or amperometric sensors [131,132]. Conductivity or capacitance sensors measure the change in conductivity or the capacitive impedance over time as a function of the concentration of the target. Potentiometric sensors measure the potential of a redox reaction in order to determine a concentration, and voltammetric sensors measure the effect of the concentration of the target on the current-potential of the redox reaction. Among these last, the amperometric sensors are a subcategory that measure the current that results from a fixed potential that is applied to a redox system and is related to the concentration of the participating species.

Table 1 summarizes recent reports of MIP-based electrochemical sensors, grouped by quantifiable electrical output generated upon the rebinding of the target molecule.

#### 3.1.1. MIP-Electrochemical Sensors in Environmental Applications

The detection and quantification of pollutants in environmental samples, such as natural waters and soils, as well as in aquatic organisms, is necessary to determine their fate and transport. Most environmental sensors have targeted endocrine disruptors, pesticides and pharmaceuticals. They occur in the environment at trace concentrations in complex matrices, which further challenges the analysis. A large number of efforts have been devoted to developing novel and economical determination and quantification alternatives. Some particularly illustrating examples are briefly described below.

A MIP sol-gel film formed by multi-walled carbon nanotubes (MWCNTs) and the conductive polymer Nafion was prepared for the determination of 2-nonylphenol, a xenobiotic endocrine disruptor [42]. The use of Nafion increased the homogeneity of the MIP sol-gel and improved the peak current of the electrochemical probe. APTES, phenyltrimethoxysilane, and a combination of these were used as monomers. A pH of 7 provided optimal electrostatic binding of the target in the MIP, due to the degree of ionization of 2-nonylphenol. The stability and repeatability tests showed a relative standard deviation of the MIP sol-gel response of only 3.6% for twenty successive measurements, and a higher current response of the electrode to the 2-nonylphenol when compared with its structural analogues. However, the effect of physical or chemical interactions in real samples, such as suspended solids, was not reported. Deng et al. [170] proposed a voltammetric sensor to detect bisphenol A, another endocrine disruptor of concern. An acetylene black paste electrode was prepared with acetylene black and paraffin and covered with 2 μL of a film of bisphenol A (10 mM), chitosan, and graphene oxide (0.25 mg/mL), using H_2_SO_4_ to crosslink the chitosan. Removal of the template was achieved by cyclic voltammetry (CV), and graphene oxide was reduced to obtain the final electrode. The best electrochemical determinations were obtained at pH 3 and with immersion of the electrode in the solution for 3 min. Six electrodes were produced to check reproducibility. The sensor was stably stored in H_2_SO_4_ at room temperature for 10 days. Repeatability was tested by using the same electrode ten times, removing the target between measurements by cyclic potential sweeps. Selectivity was good over other compounds, including various phenolic molecules, and no interference from ions was detected. Des Azevedo et al. [201] developed a MIP-hybrid electrochemical sensor for the detection of 17β-estradiol (E2) in water, based on *N*-phenylethylene diamine methacrylamide as bifunctional monomer, MAA as functional monomer, *N,N′*-diethyldithiocarbamic acid benzyl ester as initiator, EGDMA as crosslinker, and acetonitrile as porogen. However, the short linear range and low LOD would require improvements to be useful for real environmental samples. To test the sensor selectivity, 17α-estradiol (α-E2), progesterone, and (P4) estriol (E3) were used, since the three are structurally related to E2. No binding occurred between the sensor and α-E2, nor P4, showing good selectivity and specificity. However, E3 at high concentration was detected by the sensor, exhibiting a lack of selectivity due to its high structural similarity with E2.

Pesticides in environmental samples usually require arduous sample pretreatment followed by chromatographic techniques; therefore, MIPs have been proposed as a low cost, portable, and easy-to-use alternative. A MIP-based sensor for the organophosphorus pesticide methidathion was prepared using the template, the functional monomer MBA, and EGDMA in a molar ratio of 1:4:20, together with AIBN in dimethylformamide [134]. Bulk polymerization took place in an oil bath at 80 °C for 12 h, after which the monolith was ground and sieved, and the template was removed by Soxhlet extraction. The MIP particles were mixed with a TMOS sol-gel solution and deposited on a carbon screen-printed electrode (SPE). Good reproducibility and selectivity were obtained, since the sensor did not respond to other organophosphorus pesticides tested. Regeneration was achieved for five successive measurements by efficiently extracting the target with a mixture of methanol and acid. The sensor was evaluated in tap water, and although satisfactory, this relatively clean environment is far less challenging than those expected in natural water samples. Anirudhan and Alexander [145] reported a new synthesis to obtain a MIP-based potentiometric sensor to determine lindane (γ-hexachlorocyclohexane), an organochlorine pesticide, using MWCNTs. The potentiometric sensor was prepared using an active electrode of Cu and a calomel electrode as reference. First, MWCNTs were mixed with glycidyl methacrylate, which contains the epoxide functional groups essential to develop polarity in the nanotubes. Then, these MWCNTs were vinylated and polymerized with the template to obtain a covalently bonded complex matrix on their surface. The optimal pH was 3; the chlorine atoms found in the pesticide were electrostatically attracted to protonated carboxyl groups of the MWCNTs, increasing the sensitivity. The selectivity was tested using a potentiometric method and linear sweep voltammetry (LSV), with organophosphorus and organochlorine compounds, resulting in a high response compared to the other pesticides. The authors applied the sensor to the detection of lindane in fruits, vegetables, and water, but an extraction step from complex matrices was used. The sensor was able to detect the target, but the pretreatment added complexity and limited its application in situ.

Magnetic MIP nanoparticles, consisting of a magnetite core, were fabricated for the extraction, cleaning, and pre-concentration of the organophosphorus pesticide, methyl parathion in fish [178]. The nanoparticles were obtained by co-precipitation of Fe^2+^ and Fe^3+^, and a SiO_2_ shell, that were reacted with TEOS to acquire OH groups. In this way, the magnetic core-shell particles reacted with an acrylic group, obtaining active C=C groups that, in turn, were polymerized with the template in toluene. MAA, EGDMA, and AIBN were included as functional monomer, crosslinking agent, and initiator, respectively. The template was removed by Soxhlet extraction with methanol and acetic acid. The best working conditions were at pH 2 and maximum loading was reached after 1 h. Selectivity tests were performed with similar structures, confirming specific binding. The sensor could be reused for six measurements, though binding capacity was lost to some extent.

Significant efforts were directed towards the analysis of pollutants, in particular pharmaceuticals, in wastewater. Warwick et al. [138] proposed coupling MIPs with a capacitance sensor to offer a more economical alternative to the colorimetric method used in the detection of phosphates in wastewater. The selected template was phenylphosphonic acid, instead of HPO_4_^2−^ and H_2_PO_4_^−^, to avoid solubility problems, since the synthesis required organic solvents. EGDMA was the crosslinker, AIBN the initiator, and *N*-allylthiourea the functional monomer. The template and the monomer were added in a molar ratio of 2:1. Polymerization was performed under UV light for 20 min, and the MIP was ground and sieved before Soxhlet extraction of the template molecules. The sensor exhibited good performance at pH 6.5–8, emulating that of domestic wastewaters. Selectivity was studied, finding out that sulphate, nitrate, and chloride did not interfere with the detection of phosphate, thus offering stability for longer times. Reusability of the MIP membrane was achieved for up to ten times. However, the LOD and LOQ were too high, above the typical concentrations of phosphate in wastewater. *N*-formylamphetamine, an intermediate and an indicator of amphetamine synthesis, was detected in wastewaters using MIP particles that were obtained on the gold surface of a wafer electrode, with a mixture of two functional monomers, HEMA and IA, along with EGDMA, in a reaction started with AIBN at 60 °C [136]. The monomers contained methylene and carbonyl groups to bond to the phenyl and amide groups of the template. The sensor was tested in buffer solutions, but no tests in real wastewaters were performed. Zhao et al. [162] developed a MIP modified boron-doped diamond (BDD) electrode to quantitatively determine the presence of the antibiotic sulfamethoxazole in surface waters. The MIP-BDD electrode was prepared by five electropolymerization cycles using pyrrole (40 mM) as a functional monomer on a BDD electrode in the presence of the template (20 mM) at pH 7.5. The selectivity of the sensor was high; however, occurrence of sulfamethoxazole in aquatic ecosystems [205] and wastewaters [206,207] is at much lower concentrations than the detection limit. A MIP for the detection of metronidazole, a drug to prevent parasites in fish and poultry, was designed by Gu et al. [173], combining molecular imprinting with mimetic enzymes. Melamine was both the functional monomer, capable of forming hydrogen bonds, and the molecular host of the mimetic enzyme. Cu was the active center, since its complexes present enzyme-like activity; Au nanoparticles amplified the signal and casted with chitosan on a glassy carbon electrode (GCE). In parallel, CuSO_4_ in acid and melamine in water were mixed until a complex between them was formed, and then the template was included along with NaCl. After the polymer was electrodeposited on the electrode, the template was removed by ten scan cycles in Britton–Robinson buffer. Recognition and catalytic activity were successfully achieved, as well as good reproducibility and stability. Selectivity over molecules with similar electrochemical response but different in shape, size, and functional groups was good thanks to the nature of the imprinting sites; however, when the tested compound had a similar structure, the interference was greater, evidencing the lack of specificity of the MIP.

#### 3.1.2. MIP-Electrochemical Sensors in Biomedical Applications

A large number of sensors were designed with the intention of improving dose control or to measure pharmaceutical drugs in tablets, injections, or physiological fluids. However, most sensors were validated only in aqueous solutions or simulated environments, much simpler than the matrices they would encounter in biomedical applications.

Ji et al. [39] combined a MIP film with carboxylic functionalized MWCNTs GCE and Au nanoparticles to measure cholesterol concentrations. To prepare the MIP, the electrode was first immersed in a solution of the functional monomer, p-aminothiophenol, HAuCl_4_, and cholesterol to form the pre-polymerization complex, due to the strong interactions between the amino functional monomer and the acidic template. The polymer was formed through bonds between Au in the crosslinker and sulfur in the monomer, and the template was then removed by a solution of HCl in ethanol-water. Detection of the target was manifested by an increase in charge-transfer impedance, as well as a reduction in the differential pulse voltammetry current peak. The selectivity of this sensor was satisfactory and it remained stable after a month of storage at room temperature in HCl. Despite the promising results, the authors recognized that its application in clinical analysis/diagnosis would require further study. Rosy et al. [139] electropolymerized the functional monomer o-aminophenol on a GCE together with the target norepinephrine and NaClO_4_ for diagnosis and drug quality control. After the imprinting, the template was removed with H_2_SO_4_, capable of breaking the hydrogen bonds between o-aminophenol and the polymer. The sensor was tested in phosphate buffer solution (PBS) and selectivity, stability, and reproducibility were studied, with satisfactory results. A potentiometric sensor for the recognition of imidocarb dipropionate was synthesized by Rizk and coworkers [146], based on a potential difference between a MIP membrane sensor electrode and a reference electrode of Ag/AgCl. The prepolymerization solution was a mixture of the template, MAA, EGDMA, benzoyl peroxide, and acetonitrile that was bulk polymerized. Although the final application was to detect the target in the liver and kidney of animals, the sensor was only tested in aqueous solutions. A MIP sensor for the anticoagulant drug heparin was prepared using heparin as a template, MAA as a functional monomer, AIBN as an initiator, and EGDMA as a crosslinker [147]. The GCE was coated with the prepared solution and polymerization was performed under UV light. The effect of pH and prepolymerization solution formulation was tested, and the sensor was evaluated only in laboratory prepared solutions of the target. A MIP sensor for captopril, a drug used to treat hypertension, was designed using a GCE and a carbon paste electrode (CPE) [149]. The MIP particles were obtained by precipitation polymerization; captopril was dissolved in a mixture of acetonitrile and ethanol, then MAA was incorporated, followed by EGDMA and AIBN, and reacted for 12 h in an oil bath at 80 °C. The template was eluted with a solution of methanol and acetic acid. Good stability and reusability were obtained after twenty cycles of operation and selectivity over other interfering drugs was satisfactory, but tests were conducted in deionized water solutions. Li et al. [194] proposed a three-dimensional MIP modified voltammetry-based sensor for the detection of epinephrine. ZnO nanorods grew vertically on indium tin oxide (ITO) coated polyethylene terephthalate film by electrodeposition of pyrrole in the presence of the template and LiClO_4_; the template was eluted by immersion in KCl and PBS. Unfortunately, the saturation of imprinted sites prevented the linearity of the oxidation peak current vs. epinephrine in the range of 1–2000 μM. Although good selectivity facing structural analogues and repeatability were obtained, the response was not linear and the sensitivity was too low for physiologically relevant concentrations. Da Silva et al. [161] worked on a sensor to detect the antibacterial chemical norfloxacin in human urine. MWCNTs were deposited on the surface of a GCE, which was afterwards coated with a MIP film via cyclic voltammetry of polypyrrole. The human urine samples were spiked with the chemical and diluted 50% with sulfuric acid. The sensor was exposed to chemical structure analogs to the target, and interference was manifested when exposed to enrofloxacin. The MIPs were reused for thirty measurement cycles without significant change in the current response signal.

The rapid detection of biomarkers in a point of care setting is highly desirable for improved diagnosis and treatment and several authors reported efforts towards this goal. Electrochemical sensors have been reported for the detection of DNA [204] and proteins [203], although, in general, they were only tested in aqueous solutions and specificity and non-specific interactions were not explored. For example, Yola and Atar [189] developed a sensor to detect the cardiac biomarker Troponin-I in plasma. The template and pyrrole were imprinted on BN quantum dots coated GCE by cyclic voltammetry. No interference was detected due to the plasma; selectivity over other proteins in plasma, stability, and reproducibility were high. Moreira et al. [196] reported a point-of-care disposable sensor for myoglobin, another cardiac biomarker. The template and functional monomer (o-aminophenol) were adsorbed on a gold SPE and electropolymerized. The template was removed by digestion of the MIP in proteinase K that cleaved peptide bonds under mild conditions, hence avoiding alterations in the polymer. However, due to the small size of the protein, only those molecules on the outer surface could be removed, leaving the vast majority entrapped inside the film. The sensor was tested in MES buffer and synthetic urine; in this context, the results obtained were fast, sensitive, and selective. Other large molecules of interest for which MIP electrochemical biosensors have been reported are the clinical biomarkers for diagnosis of cancer and other cardiovascular diseases [208]. The imprinting of high molecular weight compounds, e.g., biomacromolecules, confronts particular additional challenges given by the size and complexity of the structure and conformation of the target, leading to binding sites with heterogeneous affinities, hindered target removal, and solvents that induce conformational changes in proteins (unfolding or denaturation) [209]. The epitope imprinting technique has been proposed to overcome these issues: in this approach, only a small but characteristic portion of the biomolecule is imprinted. However, the specificity problem may still arise if the epitope is not unique to the intended target [209].

### 3.2. Optical Sensors

Optical sensors rely on a change in an optical property, such as light absorption, fluorescence, light scattering, refractive index, or reflection, as the target rebinds to the MIP sites. This function is sufficient in the case of optically detectable targets, but, if the substance lacks optical properties, an indirect method of detection is needed. Alternatively, the change in color, fluorescence, etc., may occur after the complex formation with the MIP [210]. Different optical techniques can be used in these sensors, such as ultraviolet/visible spectroscopy (UV/Vis), fluorescence, chemiluminescence, surface plasmon resonance (SPR), and Raman scattering (RS) [211]. Conventional RS sensitivity is low because of its small cross-section, thus not attaining detection at trace level. In surface-enhanced Raman scattering (SERS), metallic nanoparticles are included (Ag, Au, etc.) to act as the active substrate where the target adsorbs with a notorious enhancement in the magnetic field [212]. Photoelectrochemical (PEC) sensors merge UV/Vis method with electrochemical sensors, by enabling amperometric detection thanks to photoirradiation. The measurements are based on electron transfer among an analyte, a semiconductor, and an electrode, coupled with photoirradiation [213]. Table 2 summarizes some reports of MIP-based optical sensors for environmental and biomedical applications.

#### 3.2.1. MIP-Based Optical Sensors in Environmental Applications

A selection of innovative MIP optical sensors is presented below, with emphasis on two main contaminant groups for the environmental field: pesticides and industrial waste. Sensors that rely on a change of color of the material upon exposure to the target compound are particularly attractive for environmental applications, as they can offer naked-eye readings in the field. However, the usual low levels of the relevant pollutant and the complex and diverse matrices encountered are major obstacles.

Dyes present in textile and paper industrial effluents are toxic to humans and the environment and require monitoring. A MIP-based evanescent wave fiber sensor for the basic red 9 (BR9) was synthesized by bulk polymerization, using 2-acrylamido-2-methyl-1-propanesulfonic acid as a functional monomer and EGDMA as a crosslinker. The amount of dye in the samples was correlated to the absorbed light in aqueous solutions [217]. However, the device was not tested in real effluents. In another approach, Duan et al. [257] proposed a sensor based on chemiluminescence resonance energy transfer in CdTe quantum dots@ luminol nanomaterials combined with chitosan/graphene oxide-magnetite-MIP for detection of chrysoidine. The CdTe QDs@ luminol amplified the chemiluminescence signal, whereas chitosan and graphene oxide improved the adsorption. When tested in the presence of coexisting substances, the sensor showed decreased specificity. A carbon dot functionalized fluorescent MIP was fabricated for the detection of dinitrotoluene in groundwater with AA as the functional monomer, EDGMA, and AIBN [247]. The sensor was tested in spiked lake water and tap water samples, with overall acceptable performance, although organic matter interfered with the fluorescence signal. This drawback was partially overcome by a non-labeled photonic MIP sensor, with the optical active structure obtained by conducting the polymerization in the pore space of a sacrificial colloidal crystal: the method allowed the detection of 2-butoxyethanol wastewater from hydraulic fracking operations. The sensor performed well, but the polyacrylic acid polymer was severely damaged by the wastewater after each use and could not be recycled [220].

Bisphenol A (BPA) is another chemical of concern that was the target of several MIP-sensors. A fluorescence MIP sensor was fabricated combining silica-coated fluorescent carbon dots via sol-gel polymerization to be used in river water samples [239]. Xue et al. [279] fabricated surface-imprinted core-shell Au nanoparticles of BPA for detection by SERS in surface water and plastic bottled beverages. Both sensors showed good performance in laboratory prepared standard solutions, and acceptable measurements in a handful of real samples, although low pH beverages resulted in very low recoveries. A photonic sensor was proposed by Kadhem at al. [221] for the detection of testosterone in natural water, another example of endocrine disrupting chemicals in the environment. A mixture of AA, EDGMA, AIBN, and the target was polymerized inside a silica particles crystal that provided the optically active morphology. Rebinding of the target produced swelling of the polymer and consequent change in the wavelength of the reflected light. The sensor showed minimal non-specific adsorption and good reusability in laboratory-made test samples.

Several optical MIP-based sensors have been reported for the detection of pesticides and veterinary antibiotics. Zhao et al. [212] fabricated a MIP for atrazine extraction from apple juice by bulk polymerization of MAA, EGDMA, chloroform, and AIBN, reacted in an oil bath at 60 °C for 24 h. The obtained monolith was ground and sieved, the template removed by Soxhlet extraction, and particles were packed into a solid-phase-extraction cartridge. The pretreated solution was analyzed by a colorimetric method based on Au nanoparticles for rapid detection by SERS, but it did not reach a low LOD nor a linearity in the response. A sensor for the herbicide 2,4-dichlorophenoxyacetic acid was developed by Wagner et al. [231] using fluorescent core-shell MIP particles in a 3-dimensional microfluidic system for droplet extraction from the water matrix and mixture with the MIP, that reached a LOD below the drinking water guideline. However, nonspecific binding due to matrix effects were observed. Electrochemiluminescent graphene quantum dots were proposed for the detection of the herbicide 2-methyl-4-chlorophenoxyacetic acid [254]. A layer of hybrid nanocomposite of graphene quantum dots and MoS_2_, in a mass ratio of 2:3, was coated on a GCE upon which the MIP was synthetized by cyclic voltammetry with 2-methyl-4-chlorophenoxyacetic acid as the selected template and the functional monomer o-PD. The template removal was accomplished by shaking in methanol and acetic acid. Samples were subjected to an extensive pre-treatment for the extraction of the target from water and food samples, and redissolution in PBS (pH 7.4). Competitive adsorption tests demonstrated good selectivity as well as good stability.

#### 3.2.2. MIP-Based Optical Sensors in Biomedical Applications

Efforts in the biomedical field are primarily aimed at developing point-of-care devices that provide non-invasive, safe, and fast detection, as well as quantification of drugs for dose control, especially when serious side effects may appear.

The detection of proteins by MIP-based sensors has been reported by fluorescence, surface plasmon resonance, and changes in the Bragg diffraction of optically active imprinted hydrogels. SPR showed good resistance to fouling and the consequent non-specific binding in biological matrices, but the technique requires relatively more expensive equipment than the measurement of Bragg diffraction. On the other hand, the response of photonic hydrogels can be affected by ionic strength or pH (buffers), possibly limiting their application to protein sensing [302].

Sensors for the biomarker α-fetoprotein were developed by Tan et al. [223] based on fluorescence and Ye et al. [278] on SERS. The fluorescent sensor was a combination of ZnS quantum dots and MIPs of functional monomers methyl methacrylate and 4-vinylphenylboronic. The two functional monomers were chosen so the boronic acid group would form a covalent bond with the template, giving a cyclic ester in alkaline medium; γ-methacryloxypropyl linked both the organic and inorganic phases, enabling the sol-gel polymerization. Serum samples were added to PBS and mixed with the MIP particles, a carbonate buffer, and, finally, diluted with water. Although high loading capacity and selectivity were obtained, the synthesis process was rather complex and the samples needed pretreatment. The SERS methodology involved Ag nanoparticles labeled MIPs with boronate affinity [278]. Specificity for α-fetoprotein was studied as well as cross-reactivity, finding out that the highest values were obtained for glycoproteins of similar molecular weight as the target. The glycoprotein RNase B was detected by a photoelectrochemical approach, given by three-dimensional anatase hierarchically cactus-like arrays vertically grown on a FTO substrate for PEC detection [291]. The electrochromic indicator employed was a Prussian blue electrode that discolored to Prussian white, as a function of the target concentration. The MIP was fabricated on TiO_2_ arrays amino-functionalized with APTES, then immersed in a solution of 2,4-difluoro-3-formylphenylboronic acid and NaBH_3_CN, washed with water and, finally, washed in a solution of NH_4_HCO_3_ at pH 8.5 containing the templates. The electrode was washed with NH_4_HCO_3_ solution and subsequently imprinted in ethanol where TEOS was added. Finally, washing with ethanol-acetic acid removed the template. The PEC measurement was performed by allowing the sample to be bound to the modified electrode, and then connecting to the Prussian blue electrode in PBS (pH 7.4). The discolored electrode was taken out and the absorbance measured after light irradiation for 10 s. Stability, selectivity, and reproducibility were studied with acceptable results.

Duan et al. [252] used chemiluminescence in the detection of dopamine, useful in the diagnosis of Parkinsonism. This work is based on silanized Fe_3_O_4_ magnetic nanoparticle-graphene oxide MIP. The magnetic graphene oxide was included in an ethanol solution of dopamine and acrylamide with EGDMA and AIBN. A solution of methanol and acetic acid was used to extract the template. A major inconvenience was caused by epinephrine, that attached to the imprinted cavities. The detection of phenylalanine in urine for diagnosis purposes was conducted by magnetic MIP nanoparticles with fluorescence spectrophotometry and RS [285]. Iron oxide nanoparticles were added to ethylene-co-vinyl alcohol-dimethylsulfoxide solution, and thereafter mixed with phenylalanine for non-covalent imprinting; the template was removed by ethanol and acetic acid. Tests on urine samples showed cross-reactivity with structurally similar compounds, in particular tyrosine and L-3,4-dihydroxyphenylalanine.

A sensor for the antipsychotic drug thioridazine was made from MIP-coated fluorescent ZnO quantum dots [240]. The quantum dots were obtained by a core precipitation from Zn(CH_3_COO)_2_ with NaOH and a silica shell. The MIP was prepared on the quantum dots by reverse microemulsion with TEOS, and NH_4_OH to hydrolyze the monomer; afterwards, the template and APTES were introduced into the solution and acetone to break the emulsion. The dots were then subjected to centrifugation and the precipitate washed with a mixture of ethanol and acetonitrile to remove the template. Tests were performed on plasma samples with a pretreatment to remove proteins and resuspension in a suitable buffer solution. Selectivity over the remaining compounds was demonstrated for the tested samples.

Significant efforts were devoted to the detection of antibiotics. A sensor for the detection of ornidazole, combining graphene quantum dots and silica MIPs, was fabricated by citric acid pyrolysis with APTES, followed by sol-gel polymerization of the target and silica matrix; methanol was used to remove the target molecules [243]. The sensor was evaluated in human plasma, pretreated to separate the proteins, and adjusted to pH 9. Repeatability, selectivity, and reproducibility were satisfactory, as well as the low interference by ions commonly found in serum. Fluorescent quantum dots were employed for the detection of sulfasalazine [244]. An amino-functionalized glass slide was incubated with APTES, covered with semi-conductor CdSeS/ZnS quantum dots, and, finally, functionalized with methacryloxypropyl trimethoxysilane to enhance adhesion. To prepare the MIP, the glass was immersed in a mixture of sulfasalazine, MAA, EGDMA, and AIBN in acetonitrile and toluene. Secondly, it was heated to 60 °C for 2 h and washed with methanol and acetic acid to remove unreacted monomers and the template. Plasma and urine samples were centrifuged and the supernatant diluted prior measurement. Reusability, selectivity over structural analogues, and reproducibility were satisfactory.

## 4. Technical Barriers to Commercialization of MIP Sensors and Devices

Molecular imprinted polymers are a promising technology in the environmental and biomedical sectors. The reported LODs comprise environmental and toxicological relevant concentrations of many chemicals of concern. Their stability and simplicity of use in comparison with more established analytical techniques make them particularly attractive for field measurements or contamination monitoring in remote areas without access to traditional chemical laboratory facilities. In the medical field, point of care diagnostics using biosensing based on enzymes and antibodies have introduced significant improvements in glucose monitoring for diabetics or pregnancy tests, providing convenience, privacy, and lower costs. MIPs have the potential to introduce similar benefits to many other conditions, since they can be fabricated for a wide range of targets, including biomarkers for cancer, infectious, and inflammatory diseases. The approach will be transformative for both medical and environmental fields; however, and in spite of the large number of patents filed worldwide on MIPs, the technology is still mostly restricted to academic laboratories [303]. Nevertheless, there is an unquestionable market need for such a device. We have conducted an extensive customer discovery study funded by the US National Science Foundation that highlighted the demand for a point of care analytical device for hormone analysis in blood from all stakeholders: health care providers, clinical laboratories, and patients.

The translation of MIP technology from the laboratory to final product has been hindered by technological challenges in two main areas: the device design and fabrication process and the scale up of the manufacturing process. A useful device should not only capture the target compound specifically from diverse and complex matrices, where most of the current research efforts have been directed, but should also provide the user with a system to read and store the measured data. The system should be small, preferably handheld, user friendly, fast, and low cost. Recent advances in electronics and optical interfaces have contributed to the solution of this problem, and interfaces for smart phones to work with MIP-based sensors have been suggested for diagnostics/analysis platforms. A fully developed and calibrated MIP-based system is still lacking, and a significant research investment will be required for its full development.

The manufacturing process, and in particular the scale up from the academic laboratory to large batches of MIP materials, is, in our view, the main obstacle to commercialization. The lack of reproducibility of MIPs between batches, both with respect to their morphological and chemical binding properties, is the main drawback. The issue seems to be related to insufficient control of fabrication parameters, which becomes extremely challenging given the complex synthesis of some of the proposed sensors. In addition, academic laboratory protocols for manufacturing usually need to be completely reengineered when shifting to large-scale manufacturing in an industrial setting, adding more research and development tasks before a successful prototype can be obtained and requiring large investments in the early stages of product development.

Finally, mass production of MIPs will consume considerable amounts of template, which can be either unavailable or economically unfeasible. A process for target recovery and purification after elution from the newly synthesized MIPs will be needed in order to reuse the target molecule in different batches.

## 5. Summary and Conclusions

The search for better sensitivity and selectivity has driven significant research in the field of materials science and engineering, and many sensors have been reported with diverse nanomaterials and sophisticated manufacturing processes. The new materials are well characterized and calibrated in laboratory made solutions, with some examples of assays in real samples. Although these attempts may improve the analytical performance, they also complicate commercialization efforts due to cost and fabrication complexity.

Currently, commercialization of MIPs is focused on niche markets in biotechnology, as well as analytical and separation chemistry. Several start-ups derived from academic laboratories are currently commercializing the technology. Semorex (Fanwood, NJ, US) specializes in protein-imprinted polymers for the elimination of specific proteins from the gastrointestinal tract in the therapy of Crohn’s disease. MIP Technologies AB (Lund, Sweden) offers tailored purification resins. AFFINISEP (Petit Couronne, France) has developed a range of solid-phase-extraction phases used in food and environment analysis, life sciences, and pharmaceuticals. MIP Diagnostics Ltd. (Bedford, UK) commercializes different types of tailor-made MIPs for in vitro diagnostics. Biotage (Cardiff, UK) designs resins for the removal of low-level contaminants, or extraction of high value desirables, from any process, particularly for the food, beverage, flavor, and fragrance industries. In addition, the life science technologies and specialty chemicals company Sigma-Aldrich (St Louis, MO, US) offers solid-phase-extraction materials based on MIP technology.

There are significant obstacles to the introduction of MIP-based sensors to the consumer market. First, the interference of structural analog compounds to the target molecule is a general problem in the literature, and some of the complex materials and synthesis processes proposed to avoid this issue are not viable for large-scale production due to the extra cost and manufacturing restrictions. A reengineering of the fabrication methods will be required in most cases, as laboratory bench protocols are converted to industrial manufacturing processes. We have reviewed numerous examples of MIP-based sensors that achieved the required LOD and linear range as needed for biological and environmental use; however, the majority of these sensors are fabricated following costly and complicated methods. In order for these materials to reach the consumer, the optimization of the production method is of utmost importance so they can be efficiently mass manufactured. Secondly, while tests in real samples are included in most of the reviewed articles, they are limited to just a few promising results. Natural waters and wastewaters are the most common matrices in environmental sensing, and their composition can vary widely in pH, dissolved solids concentration, and organic matter content. Clinical trials of MIP-based sensors are the necessary first step for regulatory approval and validation of any biomedical device. Large scale testing is an expensive and time-consuming endeavor, and constitutes one of the most important roadblocks for the advancement of the technology. Although the challenges are significant, the promises of MIP technology continue to attract numerous application-minded researchers to the field, working towards the achievement of its full potential.

## Figures and Tables

**Table 1:** MIP-based electrochemical sensors in environmental and biomedical applications.

Sensor	Functional Monomer	Electrode	Target	Sample	Linear Range	Ref.
					LOD	
Capacitance						
Aptamer-MIP	Dopamine	Au	Prostate specific antigen	TBST buffer	0.1–100 ng/mL	[133]
					1 pg/mL	
Sol-gel-MIP	*N,N*-methylene bis acrylamide (MBAA)	C	Methidathion	Wastewater	40–200 μg/L	[134]
					5.14 μg/L	
Silica NP-Au NP-MIP-Chitosan	MAA	GCE	Fumosin B_1_	Maize, milk	0.001–100 ng/mL	[135]
					0.35 pg/mL	
MIP	MAA	Au	*N*-formylamphetamine	Ultrapure water	Variable	[136]
					10 μM	
MIP	MAA	Au	Metergoline	PBS	1–50 μM	[137]
					1 μM	
MIP	*N*-allylthiourea (thiourea)	Pt	Phosphate	Wastewater	0.66–8 mg P/L	[138]
					0.16 mg P/L	
MIP	o-aminophenol	GCE	Norepinephrine	Human plasma and urine, pharmaceuticals	5 × 10^−8^–10^−5^ M	[139]
					4.9 × 10^−10^ M	
MIP	Pyrrole	PGE	Chlorpyrifos	Tap water, non-agricultural soil, corn leaves	20–300 μg/L	[140]
					4.5 μg/L	
MIP	Pyrrole	B-doped nanocrystalline diamond	Theophylline	PBS	NI	[141]
					NI	
Potentiometry						
MIP	MAA	PVC	Propranolol	Water, pharmaceuticals	10^−4^–10^−1^/10^−5^–10^−1^ M *	[142]
					10^−4^/10^−5^ M *	
MIP-Nanographene-IL	MAA	CPE	Losartan	Urine, pharmaceuticals	3 × 10^−9^–10^−2^ M	[143]
					1.82 × 10^−9^ M	
MIP NP	MAA	PVC	Histamine	Wine, fish	1.12 × 10^−6^–10^−2^ M	[144]
					1.12 × 10^−6^ M	
MIP-MWCNT	MAA	Cu	Lindane	Ground, tap, and sea water, orange, grape, tomato, cabbage	10^−9^–10^−5^; 10^−5^–10^−3^ M	[145]
					10^−10^ M	
MIP	MAA	PVC	Imidocarb dipropionate	Bovine liver and kidney	10^−5^–10^−2^ M	[146]
					2 × 10^−6^ M	
MIP	MAA	GCE	Heparine	Heparine sodium injection	3 × 10^−9^–7 × 10^−7^ M	[147]
					10^−9^ M	
MIP	4-VP	PVC	Bisphenol A	Polycarbonate drinking water bottle in water solution	0.1–1 μM	[148]
					0.02 μM	
Nanostructured MIP particles	MAA	GCE CPE	Captopril	Captopril tablet	10^−6^–10^−1^ M (GCE); 3 × 10^−9^–10^−1^ M (CPE)	[149]
					NI (GCE); 10^−9^ M (CPE)	
Voltammetry						
MIP	Pyrrole	GCE	Triacetone triperoxide	Acetonitrile	82–44,300 μg/L	[150]
					26.9 μg/L	
MIP-C NT/IL	Pyrrole	GCE	Melamine	Milk	0.4–9.2 μM	[151]
					0.11 μM	
MIP-RGO-Au NP	MAA	GCE	Carbofuran	Vegetables	5 × 10^−8^–2 × 10^−5^ M	[152]
					2 × 10^−8^ M	
MIP-MWCNT-IL-Pt	4-vinylpyridine	GCE	Tartrazine	Orange powder and soft drinks	0.03–5; 5–20 μM	[153]
					8 nM	
MIP-MWCNT	Pyrrole	GCE	Ochratoxin A	Beer, wine	5 × 10^−8^–10^−6^ M	[154]
					4.1 × 10^−9^ M	
MIP-MWCNT	Pyrrole	PGE	Triamterene	Human serum, pharmaceuticals	0.08–265 μM	[155]
					3.35 nM	
Sol-gel-MIP	Antimony-doped tin oxide (ATO)	Pt	β_2_-agonists	Human serum	5.5 nM–6.3 μM	[156]
					1.7 nM	
MIP-MWCNT	2,2′-dithiodianiline	GCE	Valganciclovir	Human serum, tablet	1–500; 500–2000 nM	[157]
					0.3 nM	
MIP	*o*-aminophenol	Au-Ag	Dopamine	Rabbit serum, rat brain tissue	2 × 10^−13^–2 × 10^−8^ M	[158]
					7.63 × 10^−14^ M	
Au NP-MIP	Au NPs@IL, ionic liquid (IL, i.e., 3-propyl-1-vinylimidazolium bromide)	GCE	Dimetridazole	Milk, honey	2 × 10^−9^–2.5 × 10^−7^; 2.5 × 10^−7^–3 × 10^−6^ M	[159]
					5 × 10^−10^ M	
MIP-GO	APTES	GCE	Bisphenol A	Milk, mineralized water	0.006–0.1; 0.2–20 μM	[160]
					0.003 μM	
MIP-MWCNT	Pyrrole	GCE	Norfloxacin	Human urine	10^−7^–8 × 10^−6^ M	[161]
					4.6 × 10^−8^ M	
MIP	Pyrrole	BDD	Sulfamethoxazole	Surface water	0.1–100 μM	[162]
					24.1 nM	
MIP-Melamine	Melamine	EPPG	8-hydroxydeoxyguanosine	Human urine	2 × 10^−8^–3 × 10^−6^ M	[163]
					3 × 10^−9^ M	
MIP-Chitosan-C dots	3-aminobenzeneboronic acid	GCE	Glucose	Human serum	0.5–40; 50–600 μM	[43]
					0.09 μM	
MIP	2-vinylpyridine, methacrylic acid, and acrylamide	CPE	Hexazinone	River water	1.19 × 10^−11^–1.1 × 10^−9^ M	[164]
					2.6 × 10^−12^ M	
MIP-Au-Polyaniline	MAA	GCE	Melamine	Milk, food	10^−5^–10 μM	[165]
					1.39 × 10^−6^ μM	
MIP-GO-Ag NP	Pyrrole	C	Bisphenol A	PVC and soil in water solution	10^−11^–10^−8^ M	[166]
					3.2 × 10^−12^ M	
MIP	MAA	CPE	Histamine	Human serum	10^−10^–7 × 10^−9^; 7 × 10^−9^–4 × 10^−7^ M	[167]
					7.4 × 10^−11^ M	
MIP-Algae	*N*-methacryloyl glutamic acid	C	Cu(II)	Human serum, soil, lake water, pharmaceutical tablet	Depending on the sample	[168]
MIP-MWNT-Au NP	*P*-aminothiophenol (p-ATP)	GCE	Cholesterol	Ethanol	10^−13^–10^−9^ M	[39]
					3.3 × 10^−14^ M	
MIP-Chitosan-Acetylene black	Chitosan	GCE	Bisphenol A	Drinking water	0.005–0.2; 0.5–10 mM	[169]
					2 nM	
MIP-Chitosan-Graphene	Chitosan (CHI)	ABPE	Bisphenol A	Bottled water, canned beverages	0.008–1; 1–20 μM	[170]
					0.006 μM	
MIP	2-aminothiophenol (2-ATP)	Au	Sodium lauryl sulfate	River and wastewater, personal hygiene products	0.1–1000 pg/mL	[171]
					0.18 pg/mL	
MIP	MAA	CPE	Methyl parathion	Soil, vegetables	10^−12^–8 × 10^−9^ M	[172]
					3.4 × 10^−13^ M	
MIP	*o*-phenylenediamine (*o*-PD)	Au	Atrazine	Deionized water	5 × 10^−9^–1.4 × 10^−7^ M	[76]
					10^−9^ M	
MIP-Cu-Melamine	Melamine	GCE	Metronidazole	Standard injection	0.5–1000 μM	[173]
					0.12 μM	
MIP-Graphene	Pyrrole	C	Tetrabromobisphenol A	Rain and lake water	0.5–4.5 nM	[174]
					0.23 nM	
Ni NP-MIP-Graphene	Pyrrole	C	Tetrabromobisphenol A	Rain, lake, and tap water	0.5–10^4^ nM	[175]
					0.13 nM	
MIP-Graphene-C NT	Pyrrole	C	Tetrabromobisphenol A	Fish	10^−11^–10^−8^ M	[176]
					3.7 × 10^−12^ M	
Ag-N-RGO-MIP	*o*-phenylenediamine (*o*-PD)	GCE	Salbutamol	Human urine, pork	0.03–20 μM	[177]
					7 nM	
Magnetite NP-MIP	MAA	mGEC	Methyl parathion	Fish	NI	[178]
					1.22 × 10^−6^ mg/L	
MIP-MWNT	MAA and vinylpyridine	GCE	Bisphenol A	Baby feeding bottle in PBS	0.1 nM–400 μM	[179]
					0.02 nM	
MIP-Ag NP	Pyrrole	PGE	Mebeverine	Human serum, capsule	10^−8^–10^−6^; 10^−5^–10^−3^ M	[180]
					8.6 × 10^−9^ M	
MIP-Graphene QD	Pyrrole	GCE	Bisphenol A	Tap and sea water	0.1–50 μM	[181]
					0.04 μM	
MIP	Amine terminated poly(*N*-isopropylacrylamide) (PNIPAAm) and *o*-phenylenediamine (*o*-PD)	Au	Folic acid	PBS	1–200 μM	[182]
					0.9 μM	
Au NP-MIP	Acetate buffer (pH 6.0), quercetin, resorcinol, methyl parathion, KClO_4_	GCE	Methyl parathion	Water, tangerine juice, sweet potato leaves	0.05–15 μM	[40]
					0.01 μM	
MIP-MWCNT	4-vinylpyridine	CPE	Bisphenol A	River, tap, and pure water	0.08–100 μM	[183]
					0.022 μM	
MIP	Aniline	PGE	L-ascorbic acid	Bovine serum, water	1–100 μM	[184]
					1 μM	
MIP-Chitosan	Chitosan	ABPE	Bisphenol A	Polycarbonate baby feeding and water bottle, PVC bottle, PVC food package in water solution	80 nM–10 μM	[185]
					60 nM	
MWCNT-Polyethyleneimine-MIP	Acrylamide	GCE	2,4-dinitrotoluene	River and wastewater	2.2 × 10^−9^–10^−6^ M	[186]
					10^−9^ M	
MIP-RGO-TiO_2_	Carboxymethyl-β-cyclodextrin (CM-β-CD)	Pt	Toltrazuril	Chicken muscles, egg	0.43–42.54 μg/L	[187]
					0.21 μg/L	
MIP-RGO	Acrylamide	PGE	D-, L-serine	Blood serum, cerebrospinal fluid, water, pharmaceutics	Depending on the sample	[188]
					0.24 ng/mL	
MIP-BN QD	Pyrrole	GCE	Cardiac Troponin-I	Human plasma	0.01–5 ng/mL	[189]
					0.0005 ng/mL	
Conductive NH_2_-MWCNT-MoS_2_-MIP	*p*-aminobenzoic acid	GCE	Sulfamerazine	Pork, chicken	3 × 10^−7^–2 × 10^−4^ M	[190]
					1.1 × 10^−7^ M	
Nano MIP-MWCNT	MAA	GCE	Trinitroperhydro-1,3,5-triazine	Tap, river, and sea water	0.1–10 nM; 0.01–1 μM	[191]
					20 pM	
MIP	*o*-Phenylenediamine (*o*-PD)	GCE	3-methylindole	Tap and lake water	0.01–1.2 μM	[192]
					4 nM	
MIP-Polydopamine-RGO	*o*-phenylenediamine (*o*-PD)	GCE	2,4-dichlorophenol	Lake water	2–10; 10–100 nM	[193]
					0.8 nM	
MIP-ZnO	Pyrrole	ITO/PET	Epinephrine	Epinephrine hydrochloride injections	1–10; 10–800 μM	[194]
					1 μM	
MIP	3-thiophene acetic acid (3-TAA)	Au	Melphalan	Pharmaceutical tablet	0.01–0.07 mM	[195]
					NI	
MIP	Vinylferrocene, 4-vinylpyridine	CPE	Benzo(a)pyrene	Ultrapure water	0–8; 2–16 μM	[78]
					0.93 μM	
MIP	Ferrocenylmethyl methacrylate	C SPE	Bisphenol A	PBS	4.7–8 nM	[79]
					3.2 nM	
MIP microbeads	Ferrocenylmethyl methacrylate	CPE	Bisphenol A	Ultrapure water	NI	[80]
					NI	
Capacitance. Voltammetry						
MIP	Aminophenol (AP)	Au SPE	Myoglobin	Synthetic human serum	1.5–4 (EIS); 0.8–3.5 (SWV) μg/mL	[196]
					1.5 (EIS); 0.8 (SWV) μg/mL	
MIP	Eriochrome black T (EBT)	Graphene SPE C SPE	Chloramphenicol	Acetonitrile buffer	1 nM–10 mM	[197]
					0.62 nM	
Amperometry						
MIP-MWCNT-Nafion	APTES and PTMS	GCE	2-nonylphenol	Tap and river water, soil	0.2–360 μM	[42]
					0.06 μM	
Au NP-MIP	Functionalized AuNPs (F-AuNPs)	Au	Dopamine	Human serum	0.02–0.54 μM	[198]
					7.8 nM	
MIP	*O*-phenylenediamine‒resorcinol	GCE	Tamoxifen	Acetate buffer	1–100 nM	[199]
					NI	
MIP	Acrylamide	Au	Norfloxacin	Human urine	10^−9^–10^−3^ M	[200]
					10^−10^ M	
Conducting MIP	*N*-phenylethylene diamine methacrylamide (NPEDMA)	Au	17β-estradiol	Ethanol/PBS solutions	10^−7^–8 × 10^−7^ M	[201]
					6.89 × 10^−7^ M	
MIP-Pt	MAA	Ti	Tetracycline	Ultrapure water	0.1–10 mg/L	[202]
					0.026 mg/L	
MIP NP	Vinylferrocene, ferrocenylmethyl methacrylate	GCE	Vancomycin	TRIS buffer	83–410 μM	[77]
					NI	
MIP	Pyrrole	Pt wire sealed in glass	Bovine leukemia virus glycoproteins	Ultrapure water	NI	[203]
					NI	
MIP	Pyrrole	PGE	DNA	Acetate buffer	NI	[204]
					NI	

*: depending on the MIP content. LOD: limit of detection. NI: not informed. ABPE: acetylene black paste. BDD: boron doped diamond. C: carbon. CPE: carbon paste electrode. EIS: electrochemical impedance spectroscopy. EPPG: edge plane pyrolytic graphite. GCE: glassy carbon electrode. GO: graphene oxide. IL: ionic liquid. ITO: indium tin oxide. ITO/PET: indium tin oxide coated polyethylene terephthalate. mGEC: magneto-electrode based on graphite–epoxy composite. MWCNT: multi-walled carbon nanotube. NP: nanoparticle. NT: nanotube. PBS: phosphate buffer solution. PGE: pencil graphite electrode. QD: quantum dot. RGO: reduced graphene oxide. SPE: Screen-printed electrode. SWV: square wave voltammetry.

**Table 2:** MIP-based optical sensors in environmental and biomedical applications.

Sensor	Form or Electrode	Functional Monomer	Target	Sample	Linear Range	Ref.
					LOD	
UV/Visible spectroscopy						
ZnFe_2_O_4_/MIP	Membrane	Acrylamide (AM)	Bisphenol A	Ultrapure water	10–1000 nM	[214]
					6.18 nM	
MIP	Membrane	Itaconic acid (IA)	Phenol	Drinking, natural, and wastewater	50 nM–10 mM	[215]
					50 nM	
MIP	Paper	MAA + polyethylenimine (PEI),	Cd(II)	Lake water	1–100 ng/mL	[216]
					0.4 ng/mL	
MIP	Particles	2-acrylamido-2-methyl-1-propanesulfonic acid (AMPSA)	Basic red 9	Ultrapure water	NI	[217]
					NI	
Magnetite-MIP	Microspheres	MAA	Rhodamine B	Wine	0.04–1.4 μg/mL	[218]
					1.05 μg/L	
MIP-Graphitic C_3_N_4_	FTO	4-vinylpyridine (4-VP)	Bisphenol A	Bottled water	5–200 μM	[219]
					1.3 μM	
MIP	Film	Acrylic acid (AA)	2-butoxyethanol	Hydraulic fracking wastewater	10 ppb–100 ppm	[220]
					3.4 ppb	
MIP	Film	AA	Testosterone	Ultrapure water	5–100 ppb	[221]
					4.2 ppb	
Magnetic MIP	NP	Triallyl isocyanurate	Sterigmatocystin	Wheat	1.8–25 ng/g	[222]
					0.63 ng/g	
Fluorescence						
MIP/Mn-ZnS QD	NP	4-vinylphenylboronic acid and methyl methacrylate	α-fetoprotein	Human serum	0.05–10 μg/L	[223]
					48 ng/L	
C dots-MIP	NP	MAA	Sterigmatocystin	Millet, rice, corn	0.05–2 mg/L	[224]
					0.019 mg/L	
MIP/Mn-ZnS QD	NP	3-aminopropyltriethoxysilane (APTES)	Nicosulfuron	River water	12–6000 nM	[225]
					1.1 nM	
MIP/POF	Capillary tube	MAA	Bisphenol A	Mineral water bottle in ethanol and water solutions	3 × 10^−9^–5 × 10^−6^ g/mL	[226]
					1.7 × 10^−9^ g/mL	
Allyl fluorescein-MIP	Microspheres	4-vinylphenylboronic acid+ MAA (VPBA/MAA)	Tetracycline	Etracycline hydrochloride, human serum, swine urine	4.26–150 nM	[227]
					4.26 nM	
MIP-IL	Microspheres	3-(anthracen-9-ylmethyl)-1-vinyl-1H-imidazol-3-ium chloride (Fluorescent IL monomer)	p-nitroaniline	River, lake, and tap water	10 nM–10 M	[228]
					9 nM	
MIP-SiO_2_-CdTe QD	Composite	Acrylamide (AM)	Sulfanilamide	River water	2–30 μM	[229]
					0.17 μM	
CdTe QD-MIP	Composite	AM	λ-cyhalothrin	River water	0.1–16 μM	[230]
					0.03 μM	
MIP	Core-shell particles	methacrylamide	2,4-diclorophenoxyacetic acid	River water	20 nM–5 μM	[231]
					20 nM	
MIP-C dots	Mesoporous structure	Amino-CDs	TNT	Tap water, soil	0.5–20 μM	[232]
					17 nM	
MIP	Hollow NP	AM	λ-cyhalothrin	Canal water	10.26–160 nM	[233]
					10.26 nM	
Fe_3_O_4_-SiO_2_-MIP	Core-shell magnetic NP	nitrobenzoxadiazole fluorophore (NBD-MA)	Rhodamine B	Methanol	NI	[234]
					10^−8^ M	
CdTe/CdS QD-MIP	Mesoporous structure	3-Aminopropyltriethoxysilane (APTES)	Diniconazole	Soil, river and wastewater	20–160 μg/L	[235]
					6.4 μg/L	
MIP	Au NC	APTES	Bisphenol A	Seawater	0.1–13 μM	[236]
					0.1 μM	
MIP	Microplate	Dansyl methacrylate	Bisphenol A	Tap, river and distilled water	10–2000 μg/L	[237]
					3 μg/L	
QD-MIP	Composite	Acrylamide (AM)	Cyphenothrin	River water	0.1–80 μM	[238]
					9 nM	
MIP-C dots	Nanocomposite	APTES	Bisphenol A	River water	10^−7^–4.2 × 10^−6^ M	[239]
					3 × 10^−8^ M	
MIP-ZnO QD	Nanocomposite	APTES	Thioridazine hydrochloride	Human plasma	4–120 nM	[240]
					0.43 nM	
MIP-C dots	Paper	MAA	3-monochloropropane-1,2-diol	Soy sauce	1–150 ng/mL	[241]
					0.6 ng/mL	
MIP-QD	Composite	APTES	Tetrabromobisphenol-A	Electronic waste	1–60 ng/mL	[242]
					3.6 ng/mL	
MIP-Graphene QD	Composite	3-aminopropyltriethoxysilane (APTS)	Omidazole	Human plasma	0.75–30 μM	[243]
					0.24 μM	
MIP-CdSeS/ZnS QD	Glass slide	MAA	Sulfasalazine	Human plasma and urine	0.02–1.5 μM	[244]
					0.0071 μM	
SiH_4_-C dots-MIP	Nanocomposite	APTES	Acetamiprid	Wastewater, apple	7–107 nM	[245]
					2 nM	
QD-MIP	Core-shell	APTES	Perfluorooctanoic acid	Tap and river water	0.25–15 μM	[246]
					25 nM	
MIP-C dots	Film	Poly(methyl acrylate-co-acrylic acid)	2,4- dinitrotoluene	Ultrapure water	1–15 ppm	[247]
					0.28 ppm	
MIP-C dots	Film	Acrylic acid(AA) + methyl acrylate (MA)	2,4- dinitrotoluene	Lake and tap water	1–15 ppm	[248]
					0.28 ppm	
MIP-QD	Nanocomposite	APTES	Thiamphenicol	Milk	0.10–100 μM	[249]
					0.04 μM	
MIP-C dots	Film	APTES	Cetricine	Urine, saliva	0.5–500 ng/mL	[250]
					0.41 ng/mL	
Chemiluminescence						
MIP/Cromatography paper	Paper strip	MAA	Dichlorvos	Cabbage leaves, tomato skin	0.003–10 μg/mL	[251]
					0.8 ng/mL	
Silanized magnetic graphene-MIP/Capillary	Capillary tube	Acrylamide (AM)	Dopamine	Urine, dopamine hydrochloride injection	8–200 ng/mL	[252]
					1.5 ng/mL	
MIP-Magnetic NP	NP	MAA	Dibutyl phthalate	Juice	3.84 × 10^−8^–2.08 × 10^−5^ M	[253]
					2.09 × 10^−9^ M	
MoS_2_-Graphene QD-MIP	GCE	o-phenyl-enediamine (o–PD)	2-methyl-4-chlorophenoxyacetic acid	Oat, tap, and lake water	10 pM–0.1 μM	[254]
					5.5 pM	
MIP-Au NP-CdSe/ZnS QD	Au electrode	Thioanilin	2-methyl-4-chlorophenoxyacetic acid	Tap, lake and river water, oat, rice	10 pM–50 μM	[255]
					2.2 nM	
MIP/Chromatography paper	Paper disk	AM	2,4-dichlorophenoxyacetic acid	Tap and lake water	5 pM–10 μM	[256]
					1 pM	
CdTe QD-Chitosan-GO-Magnetite-MIP	NP	MAA	Chrysoidine	Paper, fabric	10^−7^–10^−5^ M	[257]
					3.2 × 10^−9^ M	
MIP-Aptamer-CdS QD	SPE	Dopamine	Diethylstilbestrol	Fish	0.3–100,000 pg/mL	[258]
					0.1 pg/mL	
MIP	CPE	MAA	Azithromycin	Blood serum, urine	10^−10^–4 × 10^−7^ M	[259]
					2.3 × 10^−11^ M	
MIP-Cu nanoclusters	GCE	o-phenylenediamine	Enrofloxacin	Meat, lake water, bovine serum, human urine	0.1 nM–1 μM	[260]
					27 pM	
Surface plasmon resonance						
MIP/POF	Optical fiber	MAA	L-nicotine	Ultrapure water	1.86 × 10^−4^–10^−3^ M	[261]
					1.86 × 10^−4^ M	
MIP/POF	Optical fiber	MAA	Furfural	Transformer oil	9–30 ppb	[262]
					9 ppb	
MIP/Ag-POF	Optical fiber	Polyaniline	Ascorbic acid	Ultrapure water	10^−8^–10^−7^; 10^−6^–10^−4^ M	[263]
					1.28 × 10^−10^ M	
MIP/Ag-POF	Optical fiber	MAA	Profenofos	PBS	10^−4^ μg/L	[264]
					2.5 × 10^−6^ μg/L	
MIP	Film	Methacrylamide	Amoxicillin	Tap water, PBS	0.1–2.6 nM	[265]
					73 pM	
MIP	Film	MAA	Histamine	Fish	25–1000 μg/L	[266]
					25 μg/L	
MIP	NP	*N*-methacryloyl-(L)-histidine methyl ester	Histamine	Canned tuna, cheese	0.001–10 μg/L	[267]
					0.58 ng/L	
MIP	NP	Biotinylated phenylalanine	Prostate-specific antigen	Blood	0.001–0.2 ng/mL	[268]
					1 pg/mL	
MIP	Nanofilm	*N*-methacryloyl-(L)-tryptophan methyl ester	Carbofuran, dimethoate	River water	10–1000 ng/L	[269]
					7.11 (carbofuran); 8.37 (dimethoate) ng/L	
MIP-Au NP	Film	*N*-methacryloyl-(L)-phenylalanine	Aflatoxin B1	Corn, peanut	0.0001–10 ng/mL	[270]
					1.04 pg/mL	
MIP-Ag NP	Film	*N*-methacryloyl-(L)-histidine methyl ester	*Escherichia coli*	Mimic urine	15–1,500,000 CFU/mL	[271]
					0.57 CFU/mL	
Raman scattering						
MIP microspheres/Au-Klarite substrate	Microsphres	MAA	Nicotine	Acetonitrile	NI	[272]
					NI	
MIP/Au-Disulfide-derivatized perfluorophenylazide-Klarite substrate	NP	MAA	Propranolol	Human urine	NI	[273]
					7.7 × 10^−4^ M	
MIP/Ag dentrite nanostructure substrate	Fine particles	MAA	Melamine	Milk	0.005–0.05 mM	[274]
					0.012 mM	
Ag-MIP	Core-shell nanoplates	Methacrylamide	Rhodamine B	Ultrapure water	NI	[275]
					10^−12^ M	
Ag-MIP	Core-shell	AM	Rhodamine 6G	Water	10^−12^–10^−6^ M	[276]
					10^−14^ M	
Ag-MIP	Core-shell	MAA	4-mercaptobenzoic acid	Water	NI	[277]
					10^−15^ M	
Boronate affinity MIP-Boronate affinity SERS	Sandwich assay	APTES	α-fetoprotein	Human serum	0.001–10 μg/mL	[278]
					0.1 ng/mL	
MIP-Au NP	Core-shell	3-(triethoxysilyl)propyl isocyanate (TEPIC)	Bisphenol A	Surface water, plastic-bottled beverages	2.2 × 10^−6^–10^−4^ M	[279]
					5.37 × 10^−7^ M	
MIP-Magnetic NP	Core-shell	MAA	Ciprofloxacin	Fetal bovine serum	10^−7^–10^−4^ M	[280]
					10^−7^ M	
MIP-SiO_2_-AgNP	Core-shell	tetraethyl orthosilicate (TEOS)	Bisphenol A	Tap and lake water, milk	1.75 × 10^−11^–1.75 × 10^−6^ M	[281]
					1.46 × 10^−11^ M	
MIP-Ag	Core-shell	Acrylamide	Glibenclamide	Water	1 ng/mL–100 μg/mL	[282]
					1 ng/mL	
SiO_2_/rGO/Au-MIP	NP	Methacrylic acid, acrylamide	2,6-dichlorophenol	Water	1–100 nM	[283]
					0.02 nM	
MIP-Au NP	Core-shell	Phenyltrimethoxysilane	L-Phenylalanine	Bovine serum	10^−8^–10^−4^ M	[284]
					10^−9^ M	
UV/visible spectroscopy. Raman scattering						
MIP-Au NP	Fine particles	MAA	Atrazine	Apple juice	NA (Color); 0.005–1 mg/L (SERS)	[212]
					0.005 (Color); 0.0012 (SERS) mg/L	
Fluorescence. Raman scattering						
Magnetic MIP	NP	Poly(ethylene-co-vinyl alcohol)	Phenylalanine	Human urine	7–100 (F); 5–800 μg/mL (RS)	[285]
					NI (F); 0.4 μg/mL (RS)	
Photoelectrochemical						
MIP	ITO	Pyrrole	Bisphenol A	River and tap water	2–500 nM	[41]
					1.2 nM	
MIP-Au NP-ZnO NP	Paper	Pyrrole	Pentacholorophenol	Drinking and river water	0.01–100 ng/mL	[286]
					4 pg/mL	
F graphitic C_3_N_4_-MIP	FTO	4-vinylpyridine (4-VP)	Cr(VI)	Tap and river water	0.01–100 ppb	[287]
					0.006 ppb	
MIP/TiO_2_ NT	Thin film	o-phenyl-enediamine (o–PD)	Lindane	Drinking and river water	0.1–10 μM	[213]
					0.03 μM	
MIP/Au NP-TiO_2_ NT	Thin film	o-PD	Chlorpyrifos	Green vegetables	0.05–10 μM	[288]
					0.96 nM	
MIP/TiO_2_ NT	Vertical NT	Pyrrole	Bisphenol A	Drinking, river, and tap water, domestic sewage	4.5–108 nM	[289]
					2 nM	
MIP-TiO_2_	Nanorods	P-aminothiophenol (ATP)	Chlorpyrifos	Drinking and river water	0.01–100 ng/mL	[290]
					7.4 pg/mL	
MIP-TiO_2_	FTO glass substrate	APTES	RNase B	Human serum	0.5 pM–2 μM	[291]
					0.12 pM	
MIP-CdTe QD-Au NP	Paper/SPE	AAM	S-fenvalerate	Apple, pear, tomato, cucumber	10^−8^–10^−6^ M	[292]
					3.2 × 10^−9^ M	
MIP-TiO_2_-MWCNT	Core-shell	TiO2	Microcystin-LR	Tap, pond, and river water	1 pM–3 nM	[293]
					0.4 pM	
MIP	Porous carrier	4-vinylpyridine (VP) and *N*-isopropylacrylamide (NIPAM)	Bisphenol A	Seawater, yogurt	NI	[294]
					11.14 μg/L	
MIP	Strip/SPE	AM	Perfluorooctane sulfonyl fluoride	Tap, river, and lake water	0.05–500 ppb	[295]
					0.01 ppb	
Aptamer-MIP-GO-C_3_N_4_	FTO	Aptamer	Kanamycin	Na_2_SO_4_ solution	1–230 nM	[296]
					0.2 nM	
MIP-BiIO nanoflake array	Nanofibers	Chitosan	Triphenyl Phosphate	Tap, river, and lake water	0.01–500 ng/mL	[297]
					0.008 ng/mL	
MIP/TiO_2_ NT	Vertical NT	AM	Perfluorooctane sulfonate	Tap, river, and mountain water	0.5–10 μM	[298]
					86 ng/mL	
CdS dots-Graphene-MIP	FTO	Pyrrole	4-aminophenol	Lake water	5 × 10^−8^–3.5 × 10^−6^ M	[299]
					2.3 × 10^−8^ M	
MIP-AgI NP-BiIO nanoflake array	FTO	AM	Perfluorooctanic acid	Tap and river water	0.02–1000 ppb	[300]
					0.01 ppb	
Glass/ZnO/MIP	Nanorods	Pyrrole	Bisphenol S	PBS	2.5–12.5 μM	[301]
					0.7 μM	

LOD: limit of detection. NA: not achieved. NI: not informed. CPE: carbon paste electrode. FTO: F-doped SnO_2_. IL: ionic liquid. ITO: indium tin oxide. GCE: glassy carbon electrode. GO: graphene oxide. MWCNT: multi-walled carbon nanotube. NC: nanocluster. NP: nanoparticle. NT: nanotube. PBS: phosphate buffer solution. POF: plastic optical fiber. QD: quantum dot. rGO: reduced graphene oxide. SERS: surface enhanced Raman scattering. SPE: Screen-printed electrode.

## Data Availability

Not applicable.
